# Novel Ergot Alkaloids Production from *Penicillium citrinum* Employing Response Surface Methodology Technique

**DOI:** 10.3390/toxins12070427

**Published:** 2020-06-29

**Authors:** Memuna Ghafoor Shahid, Muhammad Nadeem, Ahmed Gulzar, Muhammad Saleem, Hafeez ur Rehman, Gul Zareen Ghafoor, Muhammad Umar Hayyat, Laila Shahzad, Rabia Arif, Rubina Nelofer

**Affiliations:** 1 Department of Botany, GC University, Lahore 54000, Pakistan; 2 Food and Biotechnology Research Center, PCSIR Laboratories Complex, Lahore 54000, Pakistan; mnadeempk@yahoo.com (M.N.); rubinanelofer@gmail.com (R.N.); 3 Department of Economics, University of Management & Technology, Lahore 54000, Pakistan; ahmedgulzar2011@gmail.com (A.G.); hafeez.rehman@umt.edu.pk (H.u.R.); 4 Department of Botany, University of the Punjab, New Campus, Lahore 54000, Pakistan; saleem.botany@pu.edu.pk (M.S.); phdgenetics@gmail.com (R.A.); 5 Sustainable Development Study Center, GC University, Lahore 54000, Pakistan; zareen.sdsc@gmail.com (G.Z.G.); umerenv@yahoo.com (M.U.H.); lailashahzad@gcu.edu.pk (L.S.)

**Keywords:** ergot alkaloids, strain improvement, UV, EMS, *Penicillium citrinum*, response surface methodology, PBD, BBD

## Abstract

Ergot alkaloids are novel pharmaceutical and therapeutic agents synthesized in this study using fungal species *Penicillium citrinum.* To get the maximum yield of ergot alkaloids a statistical process of response surface methodology was employed using surface culture fermentation technique. Initially, the strain of *Penicillium* was improved using physical (ultraviolet (UV) and chemical (ethyl methane sulfonate (EMS) treatments to get the maximum yield of ergot alkaloids through surface culture fermentation technique. After improving the strain, survival rate of colonies of *Penicillium citrinum* treated with UV and EMS was observed. Only 2.04% living colonies were observed after 150 min of exposure of *Penicillium citrinum* in UV light and 3.2% living colonies were observed after 20 min of the exposure in EMS. The mutated strains of *Penicillium citrinum* were screened for their production of ergot alkaloids and after fermentation experiments, maximum yield was obtained from PCUV-4 and PCEMS-1 strains. After strain improvement, Plackett–Burman design (PBD) and Box–Behnken design (BBD) of RSM were employed and 10-fold yield enhancement (35.60 mg/100 mL) of ergot alkaloids was achieved. This enhancement in yield of ergot alkaloids proved the positive impacts of RSM and UV on the yield of ergot alkaloids. The study provides a cost effective, economical and sustainable process to produce medically important ergot alkaloids which can be used in various pharmaceutical formulations to treat human diseases.

## 1. Introduction

Secondary metabolites are organic compounds produced by different species of bacteria, fungi and plants that are helpful in their growth, development and reproduction. They play a supportive role in the long-term impairment of an organism’s health, survival and formation of the offspring. The secondary metabolites are sometimes restricted to a specific set of species within a group of organisms. These are also helpful in the plant defense mechanism against insects and other organisms and can be used by human beings in various industrial, pharmacological and commercial applications [1]. Natural products or secondary metabolites are also known as bioactive compounds which are helping in the discovery and manufacturing of various drugs for the treatment of human ailments [2]. Many plant, animal and fungal species are known for producing naturally occurring bioactive compounds called alkaloids which are considered as a major group of naturally synthesized secondary metabolites [3].

Ergot alkaloids are being described as an assemblage of bioactive compounds of various species including plants and fungi. These were initially documented in *Claviceps purpurea* (a fungal species), the agent known for causing the disease of ergot of rye. These ergot alkaloids were the first time isolated and identified in the sclerotia produced on the kernels of rye plant. Many types of commercially and industrially significant ergot alkaloids were reported from the sclerotia of genus *Claviceps*. However, other species of fungi such as *Blansia, Epichole, Penicillium* and *Aspergillus* and several higher plants can also produce some quantity of ergot alkaloids [4]. In fungi, ascomycetes can efficiently produce alkaloids naturally and in laboratories. *Penicillium,* a well-known genus of Ascomycetes is significant for producing commercially valued secondary metabolites [5,6]. *Penicillium* species can produce significant amounts of alkaloids, antibiotics, hormones and mycotoxins [7]. Ergot alkaloids have been divided into three types on the basis of their structures such as clavines, lysergic acid amides and ergopeptines [8]. Ergot alkaloids are produced commercially for manufacturing of drugs employing various fermentation techniques. Alkaloids synthesis was regulated and enhanced industrially by adding different organic and inorganic ingredients in fermentation medium [3].

The yield of any product can also be enhanced by employing some statistical optimization parameters which help in the quick screening and selection of a number of fermentation factors/ingredients at one time to get the maximum yield of the product in fermentation medium. These statistical procedures also reflect the role and interaction of every individual factor in a specific fermentation method. response surface methodology (RSM) is composed of mathematical and statistical techniques for developing the empirical modeling of a fermentation process for optimizing conditions for producing industrially and commercially important secondary metabolites [9]. Methods and techniques in the synthesis of ergot alkaloids were improved over time with advancement of techniques and still efforts are being conceded, using the optimization techniques of fermentation technology, genetic improvement of strain and the use of protoplasts of the cultures [10].

Statistical designs such as Plackett–Burman design (PBD) [11] and Box–Behnken design (BBD) [12] are very effective and significant techniques for the investigation of targeted factors of fermentation medium. PBD is an efficient screening approach that reduces the number of experiments and gives information for the evaluation of target factors. Only the significant factors with positive responses are selected for further optimization [11,13]. In contrast, BBD is used to generate higher order responses using fewer experimental runs than a normal one factor at a time technique. The Box–Behnken design uses the twelve middle-edge nodes and three center-nodes to fit a 2nd order equation. Box–Behnken designs place points on the mid points of the edges of the cubical design region, as well as points at the center [14]. Another significance of the BBD is that it does not contain combinations for which all factors are simultaneously at their highest or lowest levels. Hence, these designs are useful in avoiding experiments performed under extreme conditions, for which unsatisfactory results may occur. Therefore, through these designs one can enhance the production of any product using fewer experiments at a low cost. Hence, the increasing demand of ergot alkaloids, because of their pharmaceutical and therapeutic nature, is compelling to establish a significant and cheaper process of the production of ergot alkaloids for commercial purposes [15].

The production of microbial ergot alkaloids cannot be achieved without the movement and permanence of the producers and this can be enhanced by inducing mutation in them. The effect of mutations on the biosynthesis of alkaloids is more reliable and applicable because of its speculative importance. The genetics of alkaloids formation has not been widely studied and the effect of mutations using physical (UV-light) and chemical (EMS) mutagens on the biosynthesis of alkaloids can achieve positive results using various strains of fungi such as *Penicillium roquefortii*, *Aspergillus niger, Trichoderma viride* [8].

In the light of the above scenario, the present study was designed after keeping in mind the significance of ergot alkaloids. The ergot alkaloids are ergotamine derivatives used to treat many ailments. They are significant in increasing the strength of uterine contractions during child birth and are used to limit postpartum bleeding. Many of the pharmaceutical companies use these derivatives and their extracts in various drugs such as for the treatment of migraine headaches as well. The main objective of the present study was the production and extraction of commercially important ergot alkaloids from fungal species using fermentation technique to contribute to the field of pharmaceutical industry. Hence, this research was designed to synthesize ergot alkaloids from fungi because the life cycle of fungi is very small than plants and it can produce large amounts of ergot alkaloids in a very short period of time. Therefore, *Penicillium citrinum* was used in the present research for the production of ergot alkaloids within a very short period of time using a sophisticated statistical technique of response surface methodology during fermentation studies. The ergot alkaloids which are produced during this study were identified as ergocryptine and ergoclavine which are very useful as pharmaceutical and therapeutic agents and can be used in various drug formulations.

## 2. Results

### 2.1. Strain Improvement

#### 2.1.1. Impact of Physical and Chemical Mutagen on *Penicillium citrinum*

The wild strain of *Penicillium citrinum* was subjected to mutation by UV irradiation and ethyl methane sulfonate (EMS) reagent.

##### Impact of UV Irradiations

**Figure 1:**
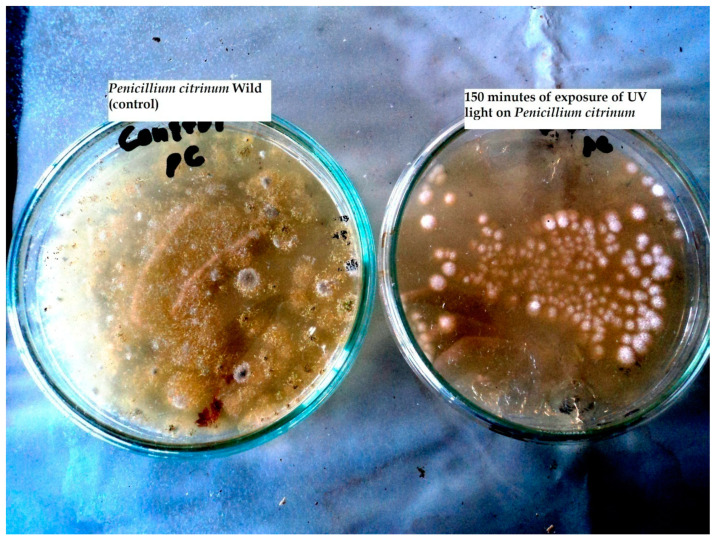
Colonies of *Penicillium citrinum* before and after exposure to UV.

The survival percentage of mutated colonies of *Penicillium citrinum* was decreased with the increase in exposure time under UV light. The minimum survival rate was found as 2.04% after 150 min of UV exposure (Table 1). The colonies survived at 150 min of exposure were grown on malt extract and agar medium as shown in Figure 1.

##### Impact of Ethyl Methane Sulfonate (EMS)

**Figure 2:**
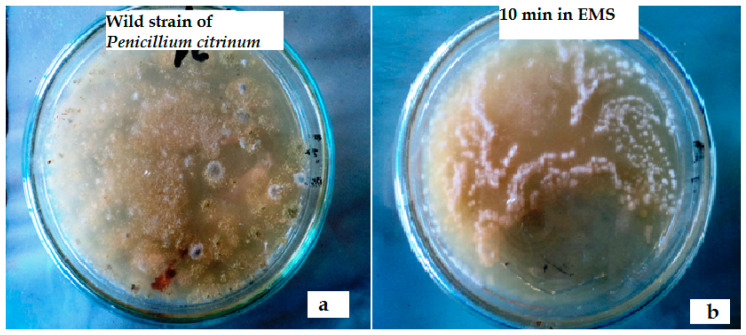
(**a**) Colonies of *Penicillium citrinum* without ethyl methane sulfonate (EMS) treatment. (**b**) *P. citrinum* colonies after 10 min of exposure in EMS.

**Figure 3:**
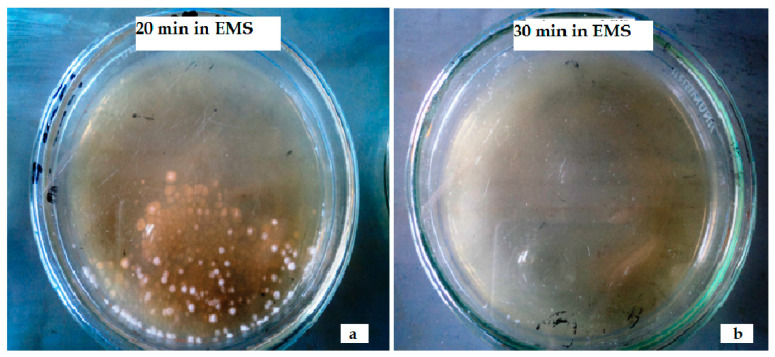
(**a**) Colonies of *Penicillium citrinum* after 20 min of exposure in EMS; (**b**) appearance of no fungal colonies after 30 min of exposure in EMS.

After exposure in EMS for variable times, the survival rate was found to be decreased rapidly and after 30 min of exposure no colony of *Penicillium citrinum* were found alive (Table 2). Minimum survival rate (3.2%) was recorded after 25 min of exposure in EMS and the presence of only a few colonies were observed (Figure 2 and Figure 3).

##### UV and EMS Mutated Strains for Ergot Alkaloids Synthesis

All of the mutated strains were screened for their ability to produce ergot alkaloids and PCUV-4 and PCEMS-3 were found as the useful mutants producing the maximum ergot alkaloids as 4.56 ± 0.01 mg/mL and 2.99 ± 0.005 mg/mL, respectively (Table 3).

### 2.2. Response Surface Methodology

The Plackett–Burman and Box–Behnken designs were used for the screening and identification of components of the fermentation medium using PCUV-4 as experimental organism.

#### 2.2.1. Screening Step Using PBD

**Figure 4:**
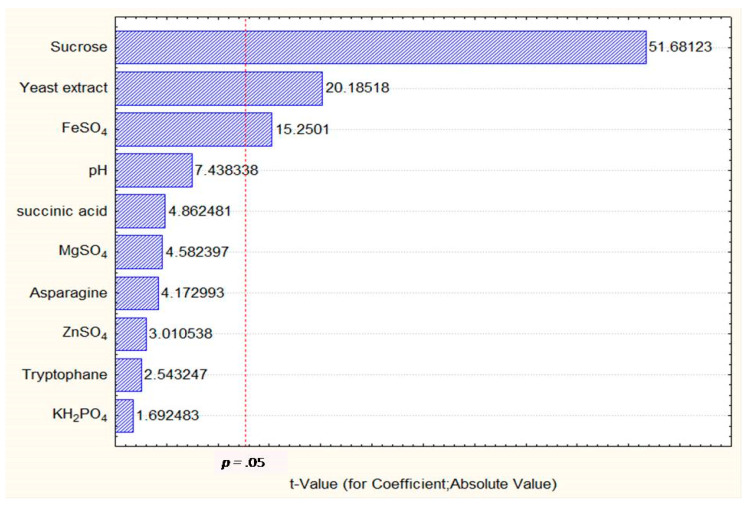
Pareto chart showing the significant variables, i.e., sucrose, yeast extract and FeSO_4_ influencing the production of ergot alkaloid yield by *Penicillium citrinum.*

Plackett–Burman design (PBD) was employed for screening of fermentation factors such as yeast extract, sucrose, asparagines, succinic acid, tryptophan, KH_2_PO_4_, MgSO_4_, FeSO_4_ and pH. The highest yield (14.74 ± 0.01 mg/mL) was achieved from run No. 2 and the lowest was observed from run No. 3 (0.36 ± 0.02 mg/mL) as given in Table 4. The Pareto chart was used to show the effect of all fermentation ingredients on ergot alkaloids production (Figure 4).

##### ANOVA for PBD Model

The ANOVA (analysis of Variance) of PBD is given in Table 5 that described the factors with *p* < 0.05 were considered significant for the production of ergot alkaloids. From Table 5, it was clearly indicated that sucrose, yeast extract and FeSO_4_ were significantly influencing the yield of ergot alkaloids. In addition, the coefficient of determination (*R*^2^) of the model was 0.9996 which explained 99.96% variance in data. Sucrose had a confidence level of above 95% in comparison to other variables and thus considered highly significant for ergot alkaloids production.

#### 2.2.2. Identification of Significant Factors Using Box–Behnken design (BBD)

Box–Behnken design (BBD) was applied to optimize the selected variables (Sucrose, yeast extract and FeSO_4_) and to find out the effect of their mutual impact on the production of ergot alkaloids. The yield of ergot alkaloids obtained from extracellular extracts of *Penicillium citrinum* ranged from 13.50 mg/mL to 35.60 mg/mL, respectively. The observed values of ergot were compared with predicted values, as presented in Table 6. Maximum ergot alkaloids yield was observed from extracellular extract of run No. 13 (35.60 mg/mL) and it was also compared with the predicted value (35.60 mg/mL). The lowest value of ergot alkaloids yield was found from run No. 6 (13.50 mg/mL) and it was almost similar to the predicted value (14.16 mg/mL) (Table 6). These values were calculated from the polynomial equation as described in Section 5.5.2.

##### ANOVA for BBD Model

**Figure 5:**
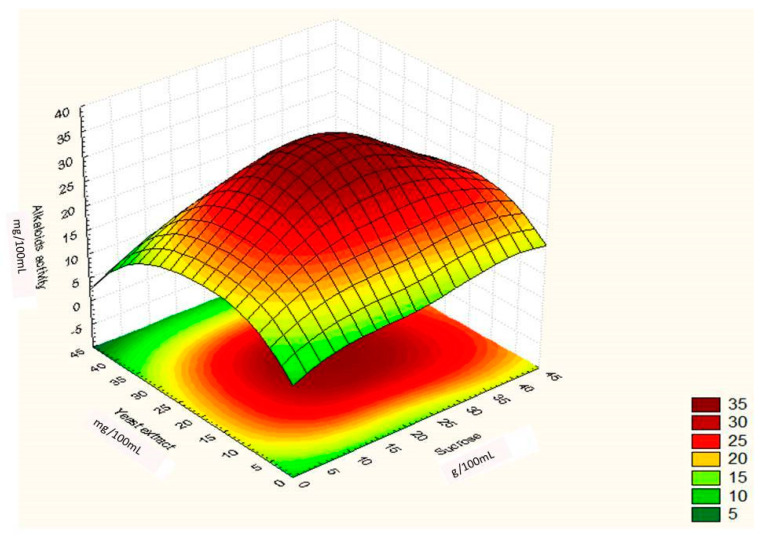
Surface graph showing interactive effect of sucrose and yeast extract on ergot alkaloid production by *Penicillium citrinum.*

**Figure 6:**
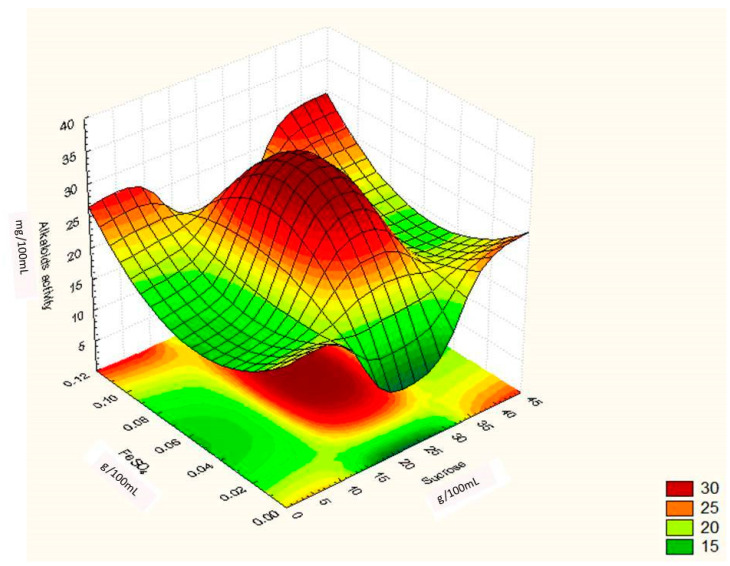
Surface graph showing interactive effect of sucrose and FeSO_4_ on ergot alkaloid production by *Penicillium citrinum.*

**Figure 7:**
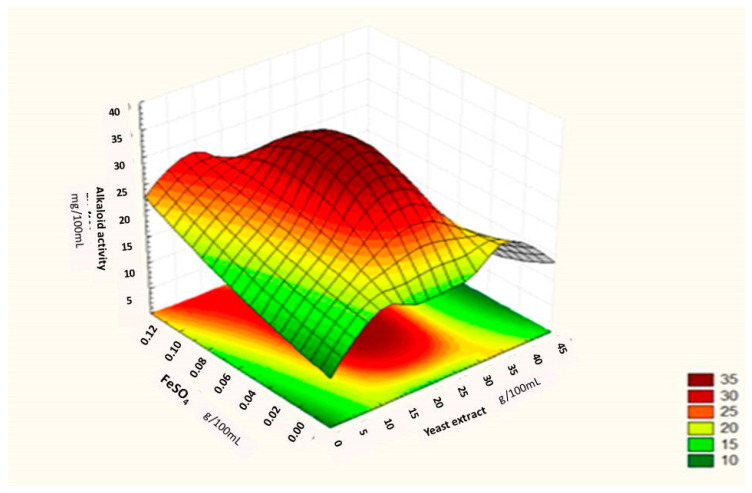
Surface graph showing interactive effect of yeast extract and FeSO_4_ on ergot alkaloid production by *Penicillium citrinum.*

The analysis of variance of the three factors (Sucrose, yeast extract and FeSO_4_) indicated that the ergot alkaloids activity can be well described by the polynomial model with a high coefficient of determination (R^2^ = 0.95). The statistical model presented in Table 7 shows that each (sucrose, yeast extract and FeSO_4_) had a significant impact on the production of ergot alkaloids. It was investigated that yeast extract remarkably influenced the production of ergot alkaloids in extracellular extracts of *Penicillium citrinum* with a value of 290.84 mg/mL. Among the combined interaction effect of these three significant variables (sucrose-yeast extract, sucrose-FeSO_4_ and yeast extract-FeSO_4_), the combination of yeast extract-FeSO_4_ interaction was found to be more significant in extracellular extract (23.52 mg/mL). Lowest yield of ergot alkaloids was obtained in the combination of “Sucrose-FeSO_4_”. Through this statistical model the insignificant interaction coefficients were eliminated, and the final polynomial equation was expressed as follows:

Y= −21.244 − 19.656 x^1^ + 3.148 x^2^ + 55.350 x^3^ − 1.791 x1^2^ – 0.02 x2^2^ + 55.350 x3^2^ − 0.019 x^1^x^2^ − 0.100 x^1^x^3^ + 0.243 x^2^ x^3^ (1)

where ‘Y’ was the predicted response, ‘X^1^, X^2^, X^3′^ were the values of sucrose, yeast extract and FeSO_4,_ respectively. Through this model it can be assumed that the model accurately represents the data in experimental region. This was confirmed by the residual analysis of the data which is not presented here. The main results of this study are presented in Figure 5, Figure 6 and Figure 7, which shows the expected ergot alkaloids production and correlation between variables in three dimensional plots.

Figure 5.shows significant additive effect of sucrose and yeast extract on the yield of ergot alkaloids. This two factor impact means that both were dependent on each other for ergot alkaloids production. The shape of the curve and the dark red color in Figure 6 also indicates that maximum alkaloids yield was obtained when 41 g/100 mL of sucrose and 39 g/100 mL of yeast extract was added in the experimental run.

Figure 6 illustrates that increasing the sucrose value at the moderate levels of FeSO_4_ in the fermentation medium led to maximum ergot alkaloids production. The optimum value deduced from Figure 6 is in accordance with the mathematically calculated optimum points. The dome shape of the curve shows the significant mutual interaction of sucrose and FeSO_4_.

In Figure 7, the additive effect of yeast extract and FeSO_4_ was found and it was observed that maximum ergot alkaloids activity was measured when fermentation medium was supplemented with 0.11 g/100 mL of FeSO_4_ and 39 g/100 mL of yeast extract. The results obtained as well as predicted by Box–Behnken design showed that a combination of 41 g/100 mL of sucrose, 39 g/100 mL of yeast extract and 0.11 g/100 mL of FeSO4 would favor maximum ergot alkaloids production (290.84 mg/mL).

##### Regression Analysis for Ergot Alkaloids Production and Comparison between the Observed and Predicted Response

**Figure 8:**
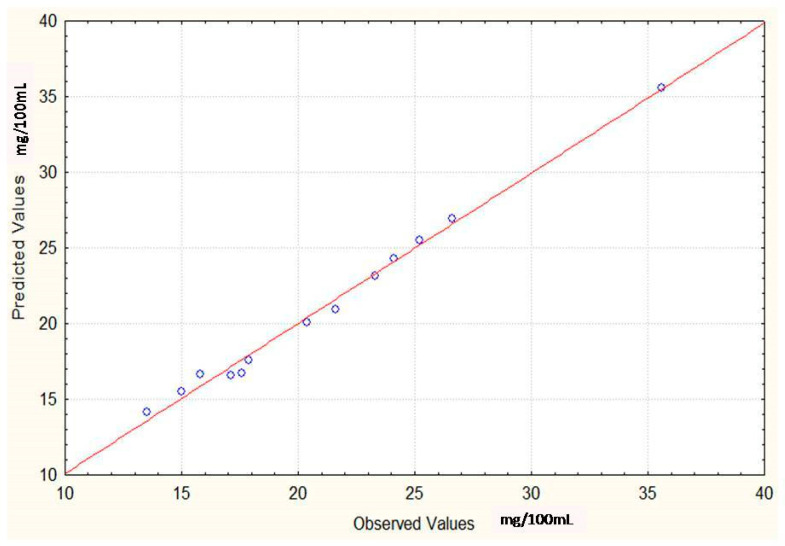
Predicted values of ergot alkaloids production by *Penicillium citrinum* using BBD.

The regression analysis was performed to predict the future response Y (ergot alkaloids yield) corresponding to the experimental data values. The regression analysis explained the difference between the observed and predicted values of the yield (Y). This was calculated by using the following equation:

e = Y − Y’(2)

where ‘e’ represents residues, ‘Y’ is the observed response or yield and ‘Y’ is the predicted response or yield of ergot alkaloids. The Figure 8 clearly indicates a non-random pattern among the predicted and observed values for the production of ergot alkaloids by *Penicillium citrinum* (PCUV-4)and a high degree of similarity was observed between the predicted (35 mg/mL)and observed response (35 mg/mL)of ergot alkaloids. It was also found that this model was a better fit model to describe the effect of optimized factors (sucrose, yeast extract and FeSO_4_) as significant independent variables on the yield of ergot alkaloids.

## 3. Discussion

Fungal organisms are considered as the depository of important secondary metabolites and many them perform significant biologic functions. These fungal species are cosmopolitan in nature and have a potential to fulfill the demand of drugs in the pharmaceutical industry [16]. These amino-alkaloids can also be obtained from higher and lower plants in lysergic acid alkaloids and gliotoxins forms [17].

In this investigation, the wild strain of *Penicillium citrinum* was subjected to physical and chemical mutagens to enhance its capability for ergot alkaloids production. The wild strain of *Penicillium citrinum* was exposed under UV irradiation and it was observed that death of the fungal species is accredited with the harmful effects of UV irradiations. Onyegeme-Okerentet al. [18] described that exposure to UV irradiation can be used for genetic variations in many species of microorganisms. This classical technique was first time used in the 1950s to synthesize maximum amount of penicillin from *Penicillium chrysogenum* Q-176. They also mutated two strains of *Penicillium chrysogenum* (UVP1 and UVP2) after UV treatment for 20 and 25 min. Veerapagu et al. [19] and Moussa [20] also concluded from their studies that with the increase in the amount of irradiations, an increase in the yield of products can be observed, but sometime the production of the fungal product can be decreased due to negative impacts of the irradiations on DNA of the organism. Ethyl methane-sulfonate (EMS) is another powerful carcinogenic mutagen that can cause significant genetic variations in microorganisms. In the present study, *Penicillium citrinum* was exposed in EMS for different time intervals. The lethality of fungal organism was found to be increased with the increase in exposure time and survival percentage of the colonies were found to be decreased (Table 2). El-Bondkly and Abeer [21] also treated *Penicillium roquefortii* with different doses of concentrated EMS and found similar results.

Furthermore, in the present study, the maximum yield of ergot alkaloids was achieved from PCUV-4 and PCEMS-3 mutant strains and their yield was also compared with the yield of wild strains of *Penicillium citrinum* (Table 3). Hamad et al. [22] also found UV irradiation to be more effective than EMS due to strong mutagenic impact of UV light on the DNA of the strain. The study also reported UV mutagenic treatments to be more efficient than the chemical treatments to get strong mutations in the DNA structure of the organism. Nadeem [23] also improved the strain by employing UV, NTG and MMS for the synthesis of alkaline proteases and found the similar results. He concluded that all physical and chemical mutagens can alter the DNA structure for enviable outcomes.

In recent studies, statistical models were reported as effective tools for the optimization of fermentation parameters in biotechnology. There are several studies for the optimization of culture conditions using these sophisticated statistical procedures [24,25]. Therefore, during the present investigation, response surface methodology (RSM) was applied as an empirical statistical technique which can be used for regression analysis of data, received from multi-factorial experimental designs. This technique is very popular for screening and optimization of various parameters of fermentation experiment [3,26]. In this investigation, Plackett–Burman design (PBD) and Box–Behnken design (BBD) were used for optimizing and screening of ingredients used to get the maximum amount of ergot alkaloids (Table 4 and Table 5). In the first step of screening the fermentation parameters, sucrose, yeast extract and FeSO_4_ were found as the most considerable ingredients as analyzed by PBD model. Wu et al. [27] screened some important ingredients of fermentation experiment for synthesizing fumigaclavine C and helvolic acid from *Aspergillus fumigatus* CY018 strain, using the same model of PBD. They also concluded that pH, amount of phosphate and the size of inoculums were considerable ingredients in the production of fumigaclavine C and helvolic acid. Rubina et al. [28] screened various fermentation medium formulations for the synthesis of a thermostable lipase using a recombinant *Escherichia coli* strain BL21. They used a similar Plackett–Burman design and found that glucose, NaCl, temperature and incubation time were the most significant variables influencing lipase production. The R^2^ value (0.979) of their study proved that PBD is highly significant model to screen the initial parameters to get the maximum lipase yield. Substantial increases in the yield of many products ranging from five to six fold using similar RSM techniques were reported by several researchers [29,30].

The next step of the present study was to apply Box–Behnken design (BBD) to optimize the yield of the product (ergot alkaloids) during fermentation studies. In this the first step was to identify the independent variables that affect the ergot alkaloids production and the next step was to study their mutual impacts on dependent response. The experiments of BBD were carried out to obtain a quadratic model consisting of 13 runs which are mentioned in Table 6 with their experimental results. Regression analysis and the analysis of variance was also performed for the three significant variables (sucrose, yeast extract and FeSO_4_). Through BBD, non-significant factors were eliminated, and the reduced model was expressed in a polynomial equation. Through the results of BBD of the present study, it can be assumed that the model accurately represents the data in the experimental region. Therefore, a great enhancement in the yield of ergot alkaloids was achieved using the fewer runs using less expensive statistical method. Lee et al. [31] applied the similar BBD in their experiments and reported an enzyme activity titer of 520 U/L for *B. thermoleovorans* using olive oil in the fermentation medium, which was higher than the activities attained by other thermophilic bacilli.

Statistical designs are significant tools which can also be used to find out the interactive influences of fermentation factors on the process performance. Therefore, during present study, the combined interactions of sucrose, yeast extract and FeSO_4_ were also analyzed using BBD. It was observed that the combination of yeast extract and FeSO_4_ is more significant for obtaining the maximum yield of ergot alkaloids from extracts of *Penicillium citrinum* (Table 6). Venil and Lakshmanaperumalsamy [14] applied the similar Box–Behnken design (BBD) for analyzing the interaction effect of temperature, (NH_4_)_2_PO_4_ and trace salts on the prodigiosin yield. They also generated a second–order polynomial equation to identify the relationship between the prodigiosin yield and three selected factors. Their fermentation medium was optimized containing 6 g/L of (NH_4_)_2_PO_4_ and trace salts (0.6 g/L) and incubation temperature was 30 °C for the enhanced production of the product. The study reported that observed responses of prodigiosin were almost equal to the predicted responses of BBD model as mentioned in the present investigation. In the end of their research, they concluded that the high correlation between the observed and predicted values of their product indicates the validity of BBD model.

Results obtained in this study are comparable to Wang et al. [32] who applied the same BBD model to find out the impact of glucose, peptones and KH_2_PO_4_ on the cell biomass formation and reported it as an efficient statistical tool. They also obtained a quadratic model and appraised the quadratic results and central points to estimate the pure process variability with chitosanase activity as response. The three components significantly affected the chitosanase activity optimized through the similar BBD model of RSM. Krishnaa et al. [33] optimized the role of pH on the biomass yield of *Borasus flabellifer* in 15 runs of fermentation experiment and concluded that BBD was the best fit model to analyze the maximum runs of experiments in a single batch. The results of the BBD model of this study are similar with Amara [34] who optimized the fermentation conditions for the synthesis of polyhydroxy butyrate by *Bacillus* species and obtained the highest yield of protease. Yasin et al. [35] applied the similar technique of RSM to enhance the flame retardant properties and low mechanical loss to fabric. They upgraded the system parameters of the finishing treatment given to the fabric using Box–Behnken statistical design. They also observed the impacts of fire resistance and other mechanical properties on fabric quality by analyzing the similar regression equation. The R^2^ estimation of the responses were above 92% which demonstrated the significance level of relationship between the predicted and experimental values of the experiment. Cheng et al. [36] optimized the nutritional medium composition required for the chitosanase production by *Streptomyces albus* YT2 and identified three significant factors (glucose, peptones and MgSO_4_) influencing the chitosanase activity by applying similar BBD statistical design. The study reported that chitosanase yield increased from 11.56 U/mL to 39.87 U/mL, which is a 3-fold increase in the yield after using the same BBD model.

Therefore, the present investigation revealed that the combination PBD and BBD for screening and optimization purpose were proved as significant and considerable designs for ergot alkaloids synthesis.

## 4. Conclusions

Ergot alkaloids are produced by many organisms and the quantity of ergot alkaloids produced by fungi is relatively less, but it can be improved by applying various techniques. Therefore, some physical and chemical mutagens can enhance the yield of the product by improving DNA of the species. In the present study, UV irradiations were proved to be useful mutagenic agent and PCUV-4 mutant was found to be the significant candidate for the production of ergot alkaloids. Ergot alkaloids were in use since many decades due to their pharmaceutical and therapeutic properties. The present investigation also concluded that response surface methodology (RSM) tools such as Plackett–Burman design (PBD) and Box–Behnken design (BBD) are more reliable statistical models to increase the yield of ergot alkaloids in a single step. It was also concluded that these fermentation studies on ergot alkaloids can contribute as an alternative and cost effective methods for the biosynthesis of the important drugs on commercial scales.

## 5. Materials and Methods

### 5.1. Microorganism and Its Maintenance

*Penicillium citrinum* was collected from the Department of Botany, GC University, Lahore Pakistan and was grown on malt extract agar (MEA) medium slants. The slants were prepared by dissolving 2 g malt extract and 2 g agar in 100 mL of distilled water in a 250 mL Erlenmeyer flask. The medium was sterilized in autoclaved at 121 °C under 15 lb/inch^2^ for 15 min. The mature culture of *Penicillium citrinum* was streaked aseptically to the slants containing 5 mL of MEA medium in a test tube. These inoculated slants were kept in incubator for 5 days at 25 °C so that they may be fully grown. The slants of *Penicillium citrinum* were subcultured after every two weeks and fully grown slants were stored at 4 °C for further analytical studies.

### 5.2. Strain Improvement

#### 5.2.1. Impact of Physical and Chemical Mutagens on *Penicillium citrinum*

The wild strains of *Penicillium citrinum* were mutated using UV irradiation and ethyl methane sulfonate (EMS) for enhancing the yield of ergot alkaloids.

##### Impact of UV Irradiations

In this step, spore suspension or stock solution of *Penicillium citrinum* was prepared in 50 mL of distilled water. The spore suspension was diluted using serial dilution method and 1 mL of the spore suspension of the last dilution was poured to sterilized Petri plates. The plates containing spore suspensions were placed under 245 nm UV lamp (purifier horizontal clean bench, Thomas Scientific, Zhejiang, China) to induce mutation for 15, 30, 45, 60, 75, 90, 105, 120, 135 and 150 min, respectively. The UV treated (spore suspension containing) Petri plates were placed overnight in dark. After 24 h, malt extract agar (MEA) medium was transferred to UV exposed Petri plates and placed in incubator at 25 °C for 10 days. After 10 days, the percentage of living colonies after mutation was compared with wild (control) colonies of strain. This was done following [37].

##### Impact of Ethyl Methane Sulfonate (EMS)

The wild strain of *Penicillium citrinum* was also exposed to Ethyl Methane Sulfonate (EMS) for inducing mutation in it. The spore suspension mixture of *Penicillium citrinum* was prepared in 50 mL distilled water and diluted by serial dilution method. After a series of dilutions, from the last dilution, 2.5 mL of spore suspension was transferred to a test tube. Then 1 mL of EMS (0.3 mL of EMS was dissolved in 0.7 mL of double distilled water to make 1 mL solution of EMS) was poured in the test tube containing the spore suspension. The mixture was shaken properly and 1 mL of it was transferred to the sterilized Petri plates. These Petri plates were exposed in EMS solution for 10, 15, 20, 25 and 30 min, respectively. After EMS exposure, malt extract agar (MEA) medium was poured in Petri plates and plates were incubated at 25 °C for 10 days. After 10 days of incubation, the survival percentage of wild and mutant colonies was noted. This method was also done after [37].

#### 5.2.2. Calculation of Survival Percentage of Colonies

Survival percentage of colonies was calculated using the following formula:

(3) $$\mathrm{Survival}\;\mathrm{rate}\;\left(\%\right)=\frac{\mathrm{No}.\;\mathrm{of}\;\mathrm{Mutated}\;\mathrm{Colonies}}{\mathrm{No}.\;\mathrm{of}\;\mathrm{Wild}\;\mathrm{Colonies}}\;\times \;100$$

### 5.3. Maintenance of Mutant Strain

The mutant strains of *Penicillium citrinum* were grown on MEA medium slants at 25 °C for 10 days. The fully grown mature slants were kept in refrigerator at 4 °C for further analyses.

#### 5.3.1. Selection of Best UV Mutant for the Production of Ergot Alkaloids

The living colonies of *Penicillium citrinum* after 135 and 150 min of UV exposure were grown on MEA medium slants and named as PCUV-1, PCUV-2, PCUV-3, PCUV-4 and PCUV-5, respectively. These mutants were provided with self-modified, optimized fermentation medium to observe the best producer of ergot alkaloids. The composition of fermentation medium is presented in Table 8.

#### 5.3.2. Selection of Best EMS Mutant for the Production of Ergot Alkaloids

The fungal colonies grown after 25 min of EMS exposure were streaked on MEA slants and named as PCEMS-1, PCEMS-2 and PCEMS-3, respectively. The self-modified optimized fermentation medium (Table 8) was used to produce the ergot alkaloids by EMS mutants. The yield by mutant strains was also compared with the yield of wild strain.

#### 5.3.3. Response Surface Methodology for Ergot Alkaloids Synthesis

In response surface methodology, statistical models such as Plackett–Burman and Box–Behnken designs were used for the statistical optimization of fermentation ingredients/variables for enhancing the yield of ergot alkaloids [14,38].

### 5.4. Preparation of Inoculum

Spore suspension of PCUV-4 (best mutant among all UV and EMS treated strains) was prepared by scratching the surface of its colonies and mixing the fungal spores in flask containing 50 mL of distilled water. The number of spores was adjusted at 10 ^6–7^ spores/mL using hemocytometer.

### 5.5. Response Surface Methodology

#### 5.5.1. Plackett–Burman design (PBD) for Screening of Fermentation Factors

The Plackett–Burman design (PBD) was used for the screening of fermentation factors (ingredients) which were used to get the highest yield of ergot alkaloids. In this step, “x” variables were screened by formulating “x + 1” fermentation factor [11]. The fermentation factors used in this step were yeast Extract, Succinic acid, Sucrose, Tryptophan, Asparagine, MgSO_4_.7H_2_O, FeSO_4_, ZnSO_4_, KH_2_PO_4_ and pH. First, a numerical factor was given to all the fermentation factors and second, all of these factors were investigated at two different levels such as −1 (low level) and +1 (high level) for the production of ergot alkaloids. The experimental range, levels and design are presented in Table 9 and Table 10.

The twelve (12) experimental runs were designed as mentioned in Table 10 after Plackett and Burman (1946) [11]. For all of the experiments, three replicates were developed and the average yield (response) of these replicates was measured. The impact of fermentation factors on the yield of ergot alkaloids was measured by formulating the following equation:

Y = β_o_ + ∑ β_Xi_ (4)

where, ‘Y’ is the yield or response, ‘β_o_’ is the intercept, ‘β_Xi_’ is the linear coefficient (fermentation factor). The 0.1-N HCl and ammonia solution were used to maintain the pH for all runs designed in PBD. All of these flasks were autoclaved at 121 °C, 15 lb/inch^2^ for 15 min and after inoculation flasks were incubated at 25 °C for 21 days.

#### 5.5.2. Identification of Significant Factors Using Box–Behnken Design (BBD)

After screening using PBD model, BBD was employed to find out the most crucial fermentation factors for the production of alkaloids. Here, further optimization of significant fermentation factors and impact of their mutual interaction on the production of ergot alkaloids was observed. This step was done after [12]. In this step, 13 runs were designed, and the significant fermentation factors were studied at three different levels such as low (−1), medium (0) and high (+1). The BBD range and levels are presented in Table 11. All the experiments were done in triplicate and the average of ergot alkaloids yield was considered as the response (Y). The following polynomial equation was formulated to identify the Y as response:

Y = β_o_ + ∑ β_ii_ + X ∑ β_ii_ x i^2^ + ∑ β_ij_ X_i_X_j_ (5)

In this ‘Y’ is the predicted response, ‘XiXj’ is the input variables which influence ‘Y’; ‘β_o_’ intercept coefficient, ‘β_ii_’ linear coefficient; ‘β_ii_ x i^2^′ quadratic coefficient and ‘β_ij_‘ interaction of these variables. ‘X1, X2 and X3′ are fermentation factors. The experimental design of BBD is presented in Table 12. The pH of all runs was maintained at 5.0 using 0.1-N HCl and ammonia solution. The media was autoclaved at 121 °C for 15 min under 15 lb/inch^2^ and after inoculation, all flasks were incubated at 25 °C for 21 days.

#### 5.5.3. Statistical Analyses of RSM

The STATISTICA version 7 (Stat-Ease, Inc., Minneapolis, MN, USA) software was used for statistical analysis and also for developing 3D figures of response surface methodology.

### 5.6. Ergot Alkaloids Determination

After 21 days of incubation the fermented broth was processed and intracellular mass (fungal mycelium) was separated from extracellular liquid (supernatant). The mycelial mass and fermented broth was stored at 4 °C for further analysis.

#### 5.6.1. Ergot Alkaloids in Fermented Broth Extract

The separated fermented broth was centrifuged at 10,000 rpm/min for 10 min at 4 °C and the extract (supernatant) was collected in a separate glass bottle. The extract was purified using rotary evaporator [39]. After purification, 1 mL of purified extract was taken in a test tube and 2 mL of Van Urk reagent was added in it. The reaction mixture was incubated at 37 °C for 30 min in water bath. The optical density (OD) was measured using Spectrophotometer (Hitachi U2900/U2910 double beam) at 590 nm.

#### 5.6.2. Ergot Alkaloids in Extract of Mycelia

The mycelia were placed in oven for drying at 40 °C for 24 h after measuring their initial weights. After 24 h, dried mycelia were soaked in chloroform for 3 h. All the soaked mycelia were crushed and grinded by sonication process at 200 rpm/min for 15 min using Ultrasonic Generator. The paste was homogenized in a homogenizer for 15 minutes. The homogenized pastes were then centrifuged at 10,000 rpm at 4 °C for 10 min. The extract of mycelia was purified using rotary evaporator. The purified extract was assayed using Van Urk reagent and measured at 590 nm in Spectrophotometer (Hitachi U2900/U2910 double beam).

## Figures and Tables

**Table 1:** Survival rate of colonies of *Penicillium citrinum* after exposure in ultraviolet (UV) light.

UV Exposure Time (min)	*Penicillium citrinum*	
	No. of Colonies	Survival Rate (%)
0	49	100
15	44	89.7
30	41	83.6
45	37	75.5
60	31	63.2
75	28	57.1
90	20	40.8
105	14	28.5
120	7	14.2
135	3	6.12
150	1	2.04

**Table 2:** Survival rate of colonies *Penicillium citrinum* in Ethyl Methane Sulfonate (EMS).

EMS Exposure Time (min)	*Penicillium citrinum*	
	No. of Colonies	Survival Rate (%)
0	31	100
10	25	80.6
15	14	45.1
20	7	22.5
25	1	3.2
30	0	0

**Table 3:** UV and EMS mutant screening.

UV treated Strains of *Penicillium citrinum*	Extracellular Extract (mg/mL)	Intracellular Extract (mg/mL)	EMS treated Strains of *Penicillium citrinum*	Extracellular Extract (mg/mL)	Intracellular Extract (mg/mL)
PCUV-1	1.49 ± 0.01	1.05 ± 0.02	PCEMS-1	1.84 ± 0.02	1.58 ± 0.01
PCUV-2	1.68 ± 0.02	1.65 ± 0.03	PCEMS-2	2.5 ±0.01	2.10 ± 0.03
PCUV-3	2.56 ± 0.05	1.65 ± 0.01	PCEMS-3	2.99 ± 0.005 *	2.78 ± 0.04 *
PCUV-4	4.56 ± 0.01 *	1.89 ± 0.03 *	Wild	2.27 ± 0.02	2.18 ± 0.02
PCUV-5	3.86 ± 0.02	1.34 ± 0.01			
Wild	2.45 ± 0.03	1.66 ± 0.01			

Each value is an average of three replicates and ± indicates the standard deviation of these replicates. And * *p* < 0.05.

**Table 4:** Screening of variables using Plackett–Burman design (PBD).

Run.	Yield of Ergot Alkaloids (mg/mL)
1	11.84 ± 0.1
2	14.76 ± 0.01 *
3	0.36 ± 0.03
4	6.53 ± 0.01
5	10.96 ± 0.01
6	11.95 ± 0.02
7	0.74 ± 0.04
8	5.38 ± 0.1
9	11.79 ± 0.03
10	7.76 ± 0.05
11	0.24 ± 0.02
12	13.02 ± 0.03

Each value is an average of three replicates and “±” indicates the standard deviation among three replicates. * *p* < 0.05.

**Table 5:** Analysis of variance for using PBD.

Source	Sum of Squares	Degree of Freedom	Mean Square	F-Value	*p*-Value
Intercept	0.46	1	0.46	8.26	0.21
Sucrose	147.78	1	147.78	2670.95	0.012
yeast extract	22.54	1	22.54	4.7.44	0.032
Succinic acid	1.30	1	1.30	23.64	0.13
MgSO_4_	1.16	1	1.16	20.96	0.14
KH_2_PO_4_	0.16	1	0.16	2.86	0.34
FeSO_4_	12.87	1	12.87	232.57	0.042
ZnSO_4_	0.50	1	0.50	9.06	0.20
Asparagine	0.96	1	0.96	17.41	0.15
Tryptophan	0.36	1	0.36	6.47	0.24
pH	3.06	1	3.06	55.33	0.085
Error	0.06	1	0.06		

**Table 6:** Observed and predicted values of yield using Box–Behnken design (BBD).

Runs	Sucrose (g/100 mL)	Yeast Extract (g/100 mL)	FeSO_4_ (g/100 mL)	Alkaloids Yield (Observed) mg/ml	Alkaloids Yield (Predicted) mg/ml
1.	41	5	0.06	22.50	21.75
2.	41	39	0.06	16.00	17.42
3.	41	22	0.01	24.55	24.32
4.	41	22	0.11	27.79	27.79
5.	5	5	0.06	18.90	17.94
6.	5	39	0.06	13.50 *	14.16 *
7.	5	22	0.01	20.40	20.06
8.	5	22	0.11	25.20	25.30
9.	23	5	0.01	16.87	16.65
10.	23	39	0.01	17.90	17.56
11.	23	5	0.11	25.20	25.53
12.	23	39	0.11	17.60	16.75
13.	23	22	0.06	35.60	35.60

Where * *p* < 0.05.

**Table 7:** Analysis of variance using BBD.

Variable	Sum of Square	Degree of Freedom	Means Square	F-Value	*p*-Value	*t*-Value
Intercept	175.21	1	175.21	167.58	0.001	−12.55
Sucrose	121.31	1	121.31	11.33	0.002	10.07
Sucrose^2^	116.22	1	116.22	119.58	0.002	−10.24
yeast Extract	294.94	1	294.94	259.87	0.000	16.12
yeast Extract^2^	308.41	1	308.41	285.67	0.000	−16.63
FeSO_4_	75.62	1	75.62	76.79	0.004	8.17
FeSO_4_^2^	49.91	1	49.91	39.89	0.006	−6.91
Sucrose, yeast Extract	3.16	1	3.16	2.01	0.251	−1.42
Sucrose, FeSO_4_	0.03	1	0.03	0.04	0.862	−0.18
yeast Extract, FeSO_4_	24.61	1	24.61	21.01	0.019	−4.58

**Table 8:** Fermentation medium for the production of ergot alkaloids by wild and mutant strains.

**Ingredients.**	**g/100 mL**
NH_4_Cl	0.2
Succinic Acid	0.5
Sucrose	5
KH_2_PO_4_	0.5
Asparagine	0.5
Tryptophan	0.5
yeast Extract	0.5
MgSO_4_. 7H_2_O	0.03
FeSO_4_	0.01
ZnSO_4_	0.002
**Fermentation Conditions**	
Incubation Time (Days)	21
Inoculum Size (ml)	5
pH	5
Incubation Temperature (°C)	25

**Table 9:** Plackett–Burman design (PBD) range and level for screening of factors.

Range and Level		Fermentation Factor
−1	+1	
5	35	Sucrose, X1
5	30	yeast Extract, X2
0.1	1	Succinic acid, X3
0.1	1	Asparagine, X4
0.1	1	Tryptophan, X5
0.1	1	KH_2_PO_4_, X6
0.25	0.625	MgSO_4_, X7
0.01	0.1	FeSO_4_, X8
0.02	0.2	ZnSO_4_, X9
3	5	pH, X 10

X1, X2……X10 are fermentation factors.

**Table 10:** PBD experimental design for screening of factors.

Runs	Variables (x)									
	X1	X2	X3	X4	X5	X6	X7	X8	X9	X10
	Sucrose	Yeast Extract	Succinic Acid	Asparagine	Tryptophan	MgSO_4_	KH_2_PO_4_	ZnSO_4_	FeSO_4_	pH
1.	35	5	0.1	0.1	1	0.625	0.1	0.2	0.1	5
2.	35	30	0.1	0.1	0.1	0.625	1	0.02	0.1	5
3.	5	5	0.1	0.1	0.1	0.25	0.1	0.02	0.01	3
4.	5	30	0.1	1	1	0.25	0.1	0.02	0.1	5
5.	35	5	0.1	1	0.1	0.25	1	0.2	0.01	3
6.	35	5	1	1	1	0.25	1	0.02	0.1	3
7.	5	5	1	0.1	1	0.625	1	0.02	0.01	3
8.	5	30	0.1	1	1	0.625	1	0.2	0.01	3
9.	35	30	1	0.1	1	0.25	0.1	0.2	0.01	5
10.	5	30	1	0.1	0.1	0.25	1	0.2	0.1	3
11.	5	5	1	1	0.1	0.625	0.1	0.2	0.1	5
12.	35	30	1	1	0.1	0.625	0.1	0.02	0.01	5

X1, X2……X10 are fermentation factors/variables.

**Table 11:** BBD experimental levels.

Level and Range			Fermentation Factor
−1	0	+1	
5	23	41	Sucrose, X1
5	22	39	yeast Extract, X2
0.01	0.06	0.11	FeSO_4_, X3

**Table 12:** BBD experimental design for optimization of fermentation factors.

Runs	Variables		
	X1	X2	X3
	Sucrose	Yeast Extract	FeSO_4_
1	41	5	0.06
2	41	39	0.06
3	41	22	0.01
4	41	22	0.11
5	5	5	0.06
6	5	39	0.06
7	5	22	0.01
8	5	22	0.11
9	23	5	0.01
10	23	39	0.01
11	23	5	0.11
12	23	39	0.11
13	23	22	0.06
